# Management of Acute Iliofemoral Deep Venous Thrombosis in Inflammatory Bowel Disease – Transitioning From Conservative Management to Early Intervention

**DOI:** 10.7759/cureus.17426

**Published:** 2021-08-25

**Authors:** Arshpreet Singh Badesha, Kajal Kaur Tamber, Katarzyna Powezka, Stacie Hodge, Taha Khan

**Affiliations:** 1 Medical School, University of Manchester, Manchester, GBR; 2 Vascular and Endovascular Surgery, The Royal Oldham Hospital, Oldham, GBR; 3 Vascular and Endovascular Surgery, Manchester Vascular Service, Manchester, GBR

**Keywords:** deep vein thrombosis (dvt), deep venous stenting, catheter-directed thrombolysis, endovascular procedures, inflammatory bowel disease

## Abstract

Deep vein thrombosis (DVT) is a frequent complication in patients suffering from inflammatory bowel disease (IBD), especially in those with frequent relapses of the disease or extensive inflammatory lesions. The aetiology for the increased risk is multifactorial.

Current evidence on management of acute iliofemoral DVT in IBD patients is scarce. This case series highlights two cases of active IBD, who developed acute iliofemoral DVTs and were treated with catheter-directed thrombolysis (CDT).

This report demonstrates that CDT is effective in clearing the clot burden and producing significant symptomatic improvement in the absence of major complications. An individualised approach must be taken for the management of IBD patients with acute iliofemoral DVT.

## Introduction

Inflammatory bowel disease (IBD) is a hypernym for conditions causing chronic immune-mediated inflammation of the gastrointestinal tract, incorporating ulcerative colitis (UC) and Crohn’s disease. Venous thromboembolism (deep vein thrombosis [DVT] and pulmonary embolism) is an extra-intestinal manifestation of IBD; the risk of venous thromboembolism (VTE) is three-fold higher in IBD patients in comparison to the general population [1]. VTE is more common in patients with active disease, frequent relapses, pancolitis, and colonic involvement in Crohn’s disease [2]. The multifactorial aetiology of DVT involves activation of the coagulation cascade and platelet aggregation secondary to the release of inflammatory cytokines [3]. In patients with IBD, the balance between procoagulants and profibrinolytic factors determines plasma coagulability [4].

Post-thrombotic syndrome (PTS) is a recognised complication of DVT and manifests as a spectrum of symptoms ranging from mild leg discomfort to venous ulceration [5]. PTS can impair physical mobility [6], contribute to health-related anxiety [7], and result in significant absenteeism from employment [8]. Patients with DVT undergo immediate anticoagulation therapy, early mobilisation, and may receive compression hosiery to prevent thrombus progression, improve venous outflow, and reduce the risk of PTS as per the European Society of Vascular Surgery guidelines [9].

Early thrombus resolution improves long-term outcomes by restoring patency and preserving endothelial function [10]. This is particularly significant in IBD patients with iliofemoral thrombosis who have a significant burden of chronic disease. Early iliofemoral venous recanalisation can be achieved through thrombolysis (catheter-directed or pharmaco-mechanical) or by mechanical thrombectomy (e.g. ClotTriever®, Inari Medical, CA, USA) [11-13]. Patients may then need venoplasty and stenting to maintain deep vein patency if any significant stenosis or occlusion is identified [14].

Despite extensive research into the treatment of acute and chronic iliofemoral DVT, there is currently no published literature on DVT management in IBD patients. Given that IBD increases the risk of VTE, it is important to explore the treatment approaches in these patients. This case series discusses two IBD patients who underwent successful management of acute iliofemoral DVT.

## Case presentation

Case 1 - 57-year-old female with a background of UC

Background

**Figure 1:**
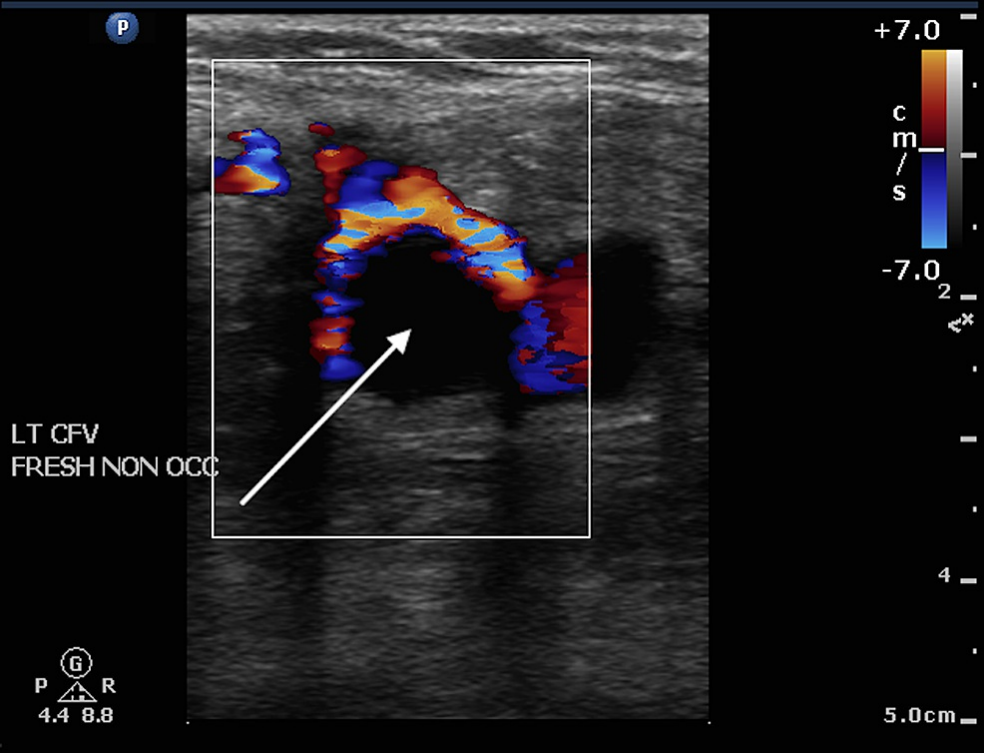
Scan on admission – venous duplex scan White arrow: thrombus in the common femoral vein

**Figure 2:**
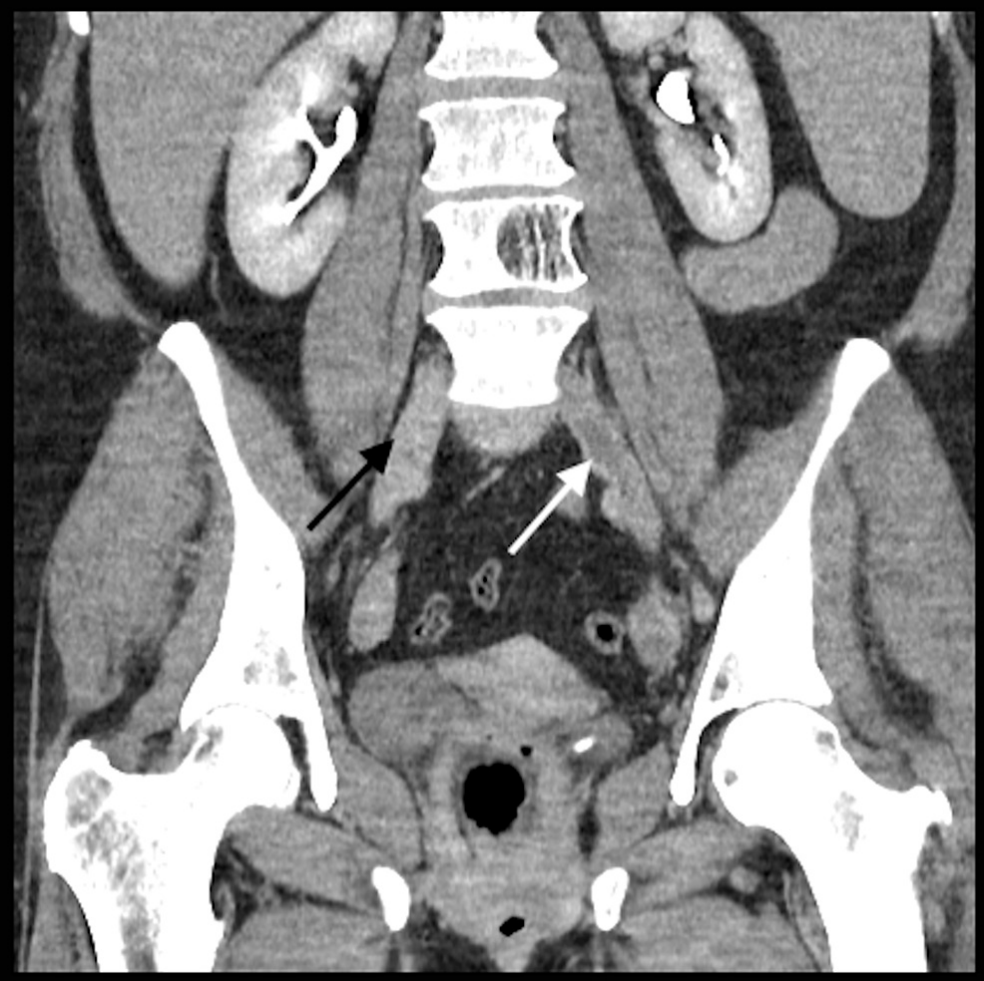
Scan on admission – CT venogram (coronal view) White arrow: thrombus in the left common iliac vein; black arrow: patent right common iliac vein

This patient presented with a six-day history of left leg swelling and discomfort. Duplex sonography and computerised tomography confirmed the presence of thrombus extending from the left common iliac vein to the left distal external iliac vein and into the left common femoral vein (Figure 1 and Figure 2).

Surgical Management

**Figure 3:**
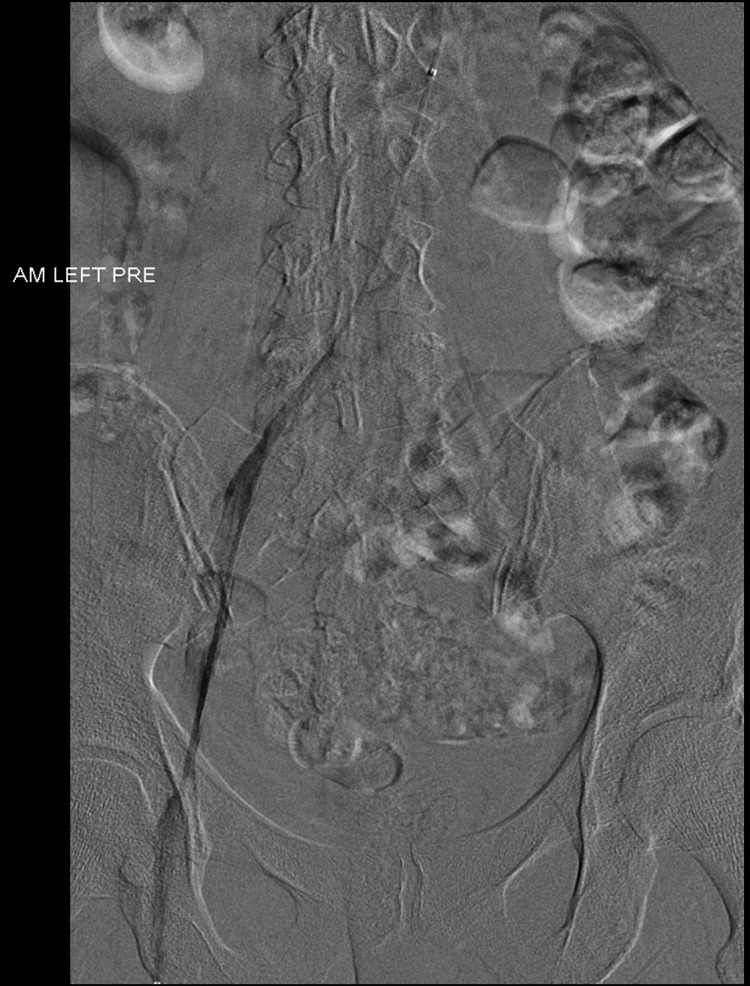
Pre-thrombolysis (prone position) venogram of the left iliofemoral segment

**Figure 4:**
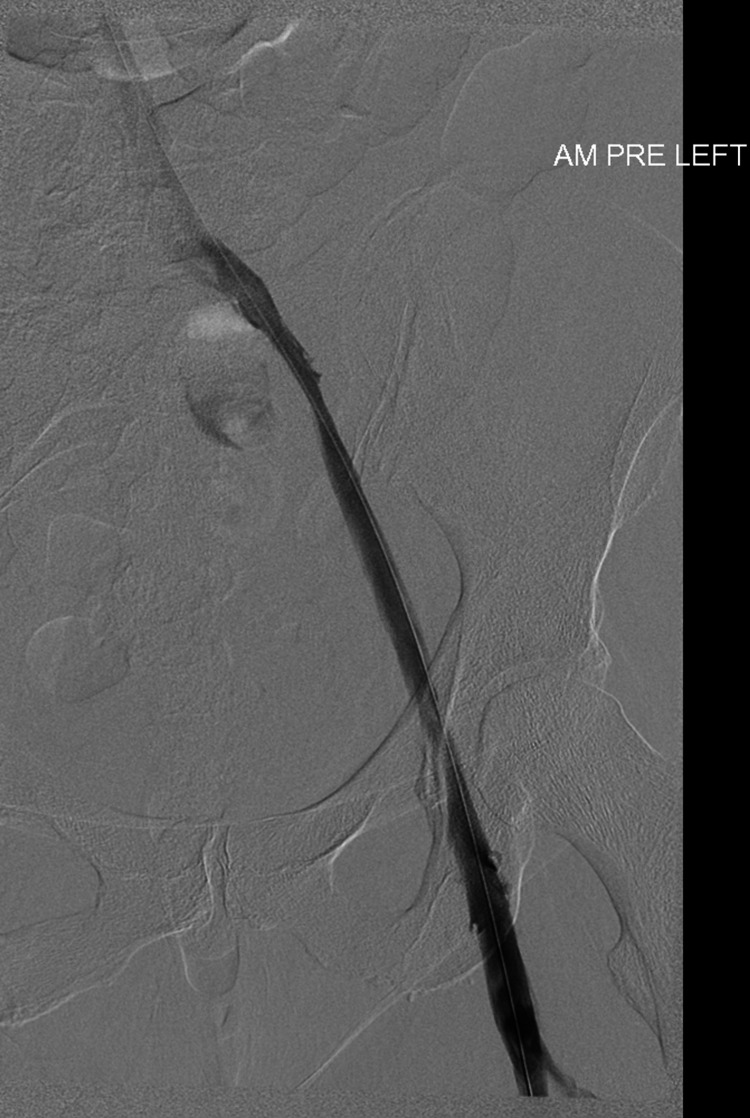
Post-thrombolysis (supine position) venogram of the left iliofemoral segment demonstrating complete dissolution of the thrombus following 72 h of catheter-directed thrombolysis

The patient received catheter-directed thrombolysis (CDT) using a 5F Fountain® catheter (Merit Medical Systems, UT, USA) positioned in the thrombus via a left popliteal vein puncture. Recombinant tissue plasminogen activator (r-tPA) was infused at a rate of 1 mg/h; after 72 h there was complete clearance of the thrombus (Figure 3 and Figure 4).

**Figure 5:**
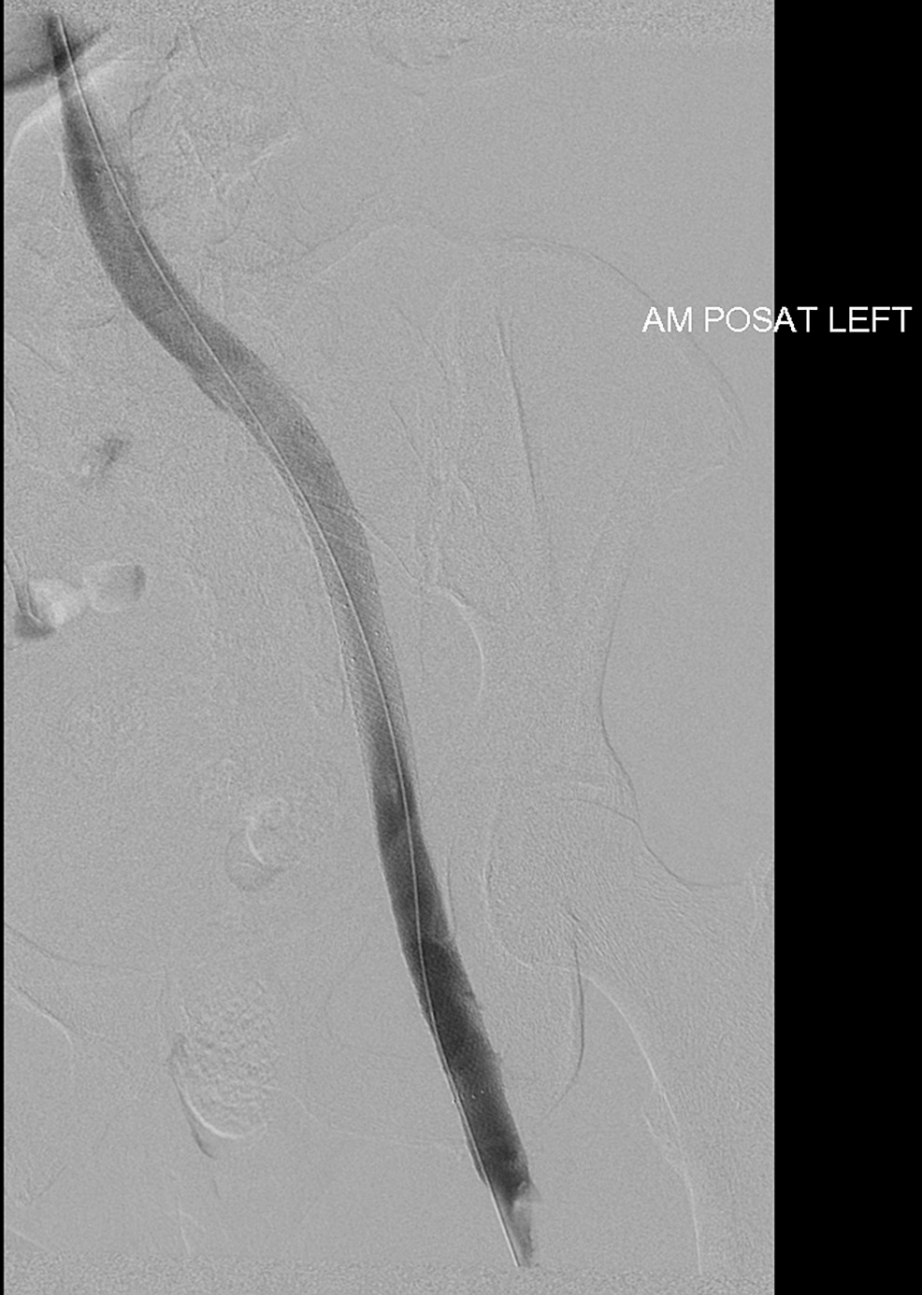
Left iliac segment post-stenting venogram

Final venography identified left common iliac vein and distal left external iliac vein stenoses. Therefore, venoplasty was performed at 18 atm using a 14 mm x 40 mm Atlas® balloon (Bard Peripheral Vascular, Temple, AZ, USA). The left iliofemoral segment was stented using closed cell stents (two 14 mm x 120 mm Vici Venous Stents® (Boston Scientific, Marlborough, MA, USA) with a bridging 14 mm x 90 mm Vici Venous Stent® (Figure 5). 

Post-operative Recovery and Surveillance

The patient was initiated on therapeutic low-molecular-weight heparin (12-hourly 1 mg/kg enoxaparin) for six weeks with a view to transitioning to long-term warfarin (international normalised ratio range 2.0-3.0) alongside thigh-length class II compression hosiery. Post-operative duplex sonography the following morning demonstrated patent stents with good flow. Surveillance sonography was initiated with scans at two weeks, six weeks, three months, six months, 12 months, and annually thereafter.

Post-stenting Complications

Five months following stent deployment, the patient presented with a refractory flare of UC necessitating a subtotal colectomy. Duplex sonography performed during the admission revealed an in-stent stenosis. Venoplasty (18 atm, 14 mm x 40 mm Atlas® balloon) was performed prior to the colectomy to reduce the risk of post-operative DVT and in-stent thrombosis. The procedure was successful and follow-up sonography revealed patent stents with no residual thrombus. The patient experienced no major complications and was continued on the post-stenting surveillance programme (previously described). The stents remained patent after 19 months.

Case 2 - 41-year-old male with a background of Crohn’s disease

Background

**Figure 6:**
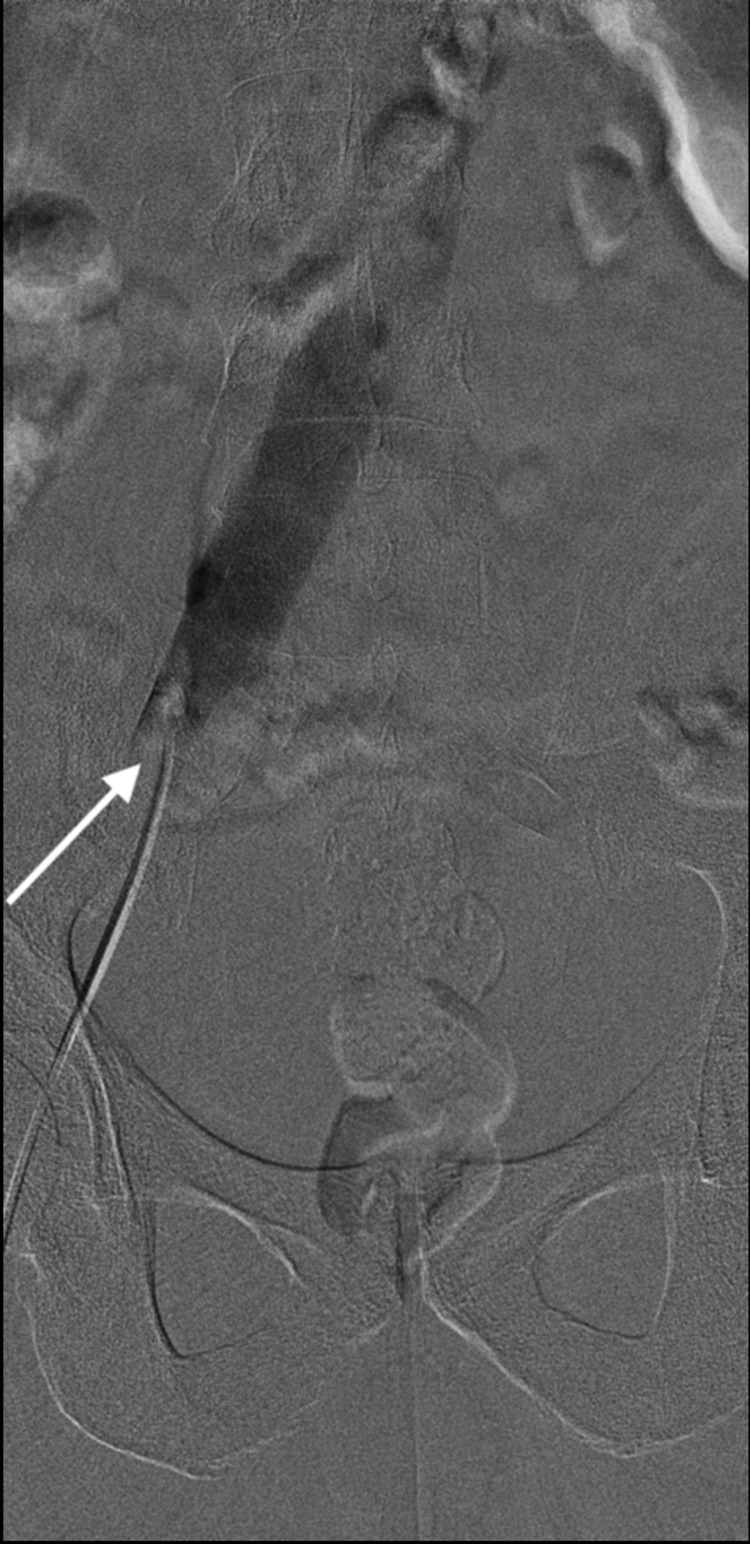
Venogram confirming an occluded external iliac vein (white arrow)

**Figure 7:**
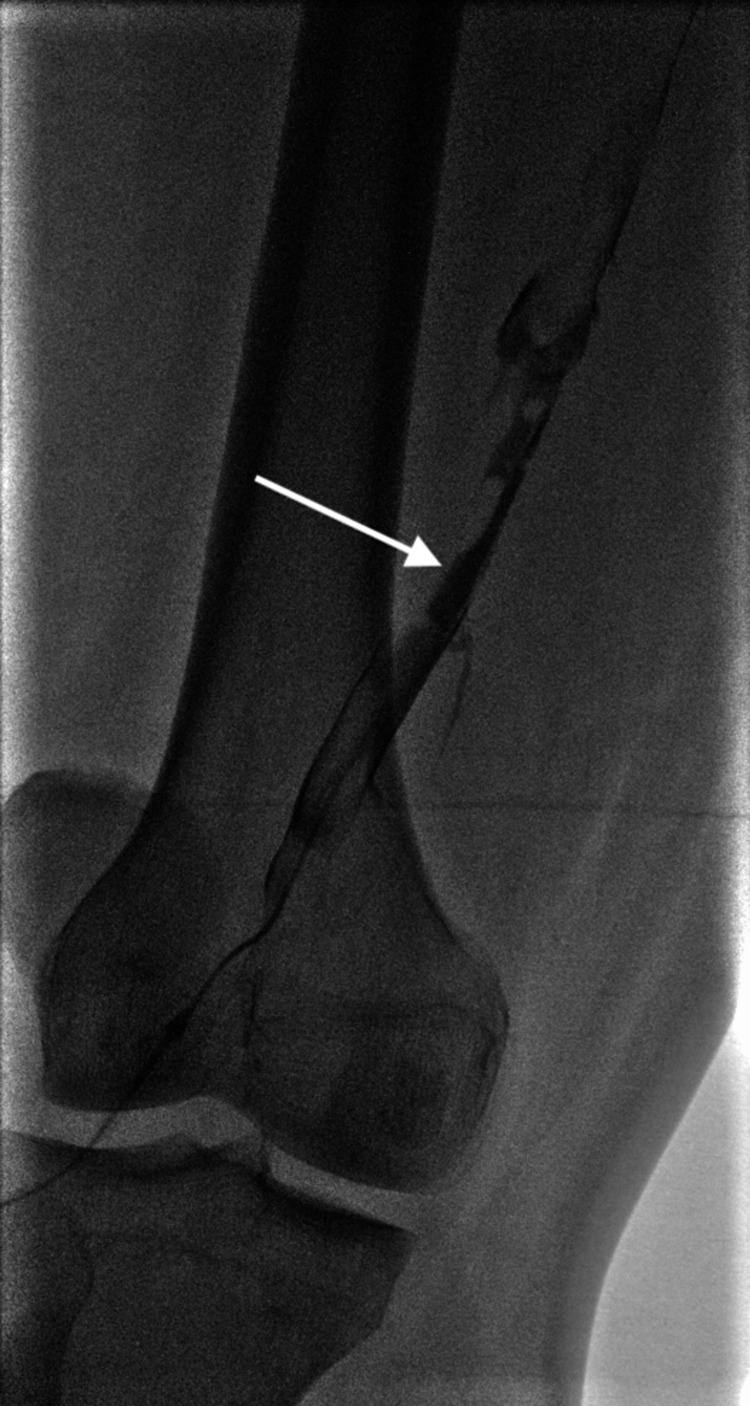
Venogram demonstrating a non-occlusive thrombus in the superficial femoral vein (white arrow)

This patient presented with a 10-day history of left leg swelling and pain. Duplex sonography and venography confirmed occlusion from the left common iliac vein to the left common femoral vein (Figure 6 and Figure 7).

Surgical Management and Post-operative Recovery

The patient received CDT using r-tPA (infused at a rate of 1 mg/h) via a 5F Fountain® catheter positioned in the thrombus, accessed via the left popliteal vein. Following 72 h of treatment, the thrombus cleared.

**Figure 8:**
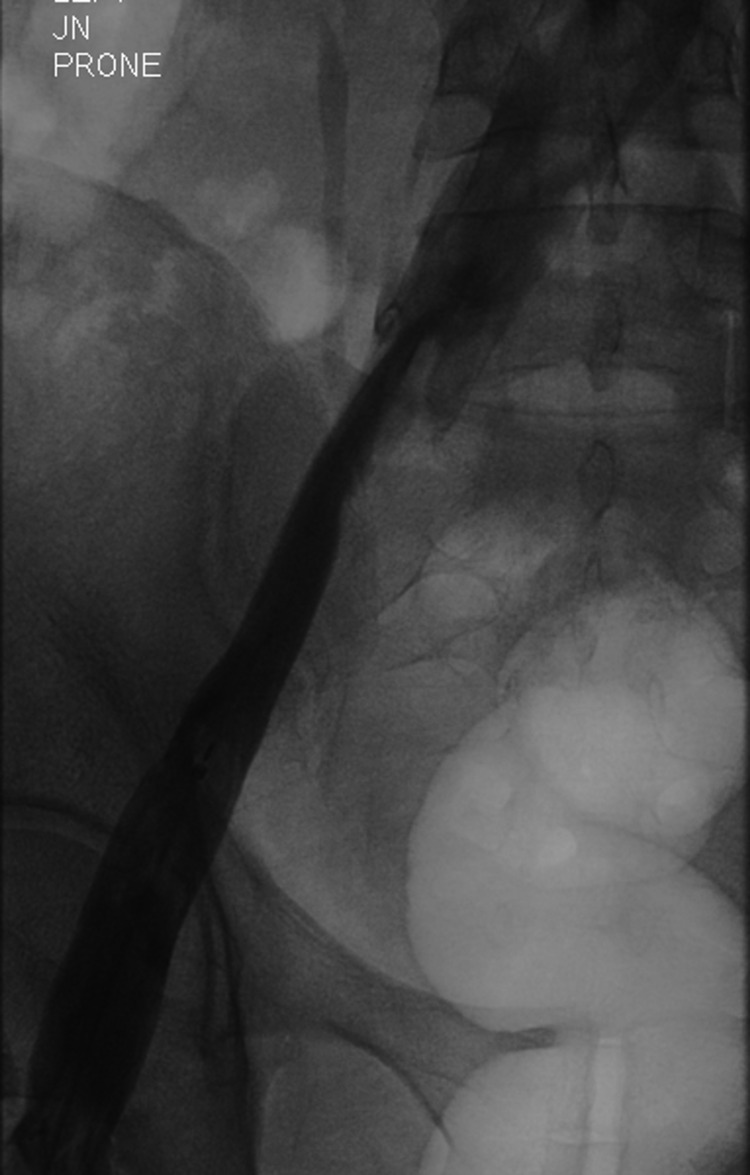
Venogram from the third day of catheter-directed thrombolysis demonstrating patent external and common iliac veins with contrast filling to the inferior vena cava

Post-operative venography confirmed improved venous flow with no visible collateral veins and therefore stenting was not required (Figure 8). There were no major complications following CDT and the patient experienced full symptom resolution. The patient was discharged on apixaban (10 mg twice daily for seven days, then 5 mg twice daily for six months). This post-operative management differed from case 1 as due to the absence of deep venous stenting, intensive long-term anticoagulation was not required.

## Discussion

Lower-limb DVT is a recognised extraintestinal manifestation of IBD [1]. Evidence on the management of proximal acute iliofemoral DVT in patients with IBD is sparse, with only case reports published within the literature [2,15]. To date, this is the first case series on endovenous management of acute iliofemoral DVT in patients with IBD.

Both cases highlight the effectiveness of thrombolysis in treating acute iliofemoral DVT and achieving significant symptomatic improvement. Furthermore, the treatment did not result in major haemorrhage, which is a concern in IBD patients. Nonetheless, it is important to be aware of thrombolysis-related complications such as minor and major bleeding, pulmonary embolism, and puncture site complications [16].

Traditional management of acute iliofemoral DVT is oral anticoagulation for 3-6 months [9]. However, this has limited influence on vessel recanalisation, and, therefore, does not reduce the incidence of venous fibrosis, valvular damage, or the development of chronic venous insufficiency.

Meta-analysis data have demonstrated that the incidence of PTS is lower following CDT in comparison to oral anticoagulation (odds ratio [OR]: 0.32; 95% confidence interval [CI]: 0.12-0.85, p = 0.022) [17]. This is further supplemented by guidance from the American College of Chest Physicians, American Heart Association, and the Society of Interventional Radiology who recommend CDT as an adjunct to anticoagulation rather than a standalone therapy [18], particularly in younger patients without major comorbidities and a long life-expectancy [12,19,20].

This case series demonstrates that thrombolysis is safe in the management of acute iliofemoral DVT in IBD patients and subsequent prevention of PTS. Prevention of PTS is paramount and can be achieved with effective thrombus clearance, which is regularly not achieved with medical management alone, particularly in IBD patients. Therefore, thrombolysis with or without deep venous stenting may be considered as a first-line treatment in suitable patients with IBD complicated by iliofemoral DVT.

Vascular surgery units treating iliofemoral DVT should have an established protocol for CDT, a robust follow-up program, and services for the management of complications encountered in short- and long term after thrombolysis. Pathways that enable closer cooperation between gastroenterology specialists and vascular surgeons would facilitate the on-going care for IBD patients. Nonetheless, the decision regarding invasive surgical management of iliofemoral DVT should be made on an individual basis using a multidisciplinary team approach, taking into consideration the specific risks to a given individual and providing patients with the necessary information to enable them to make an informed decision. 

## Conclusions

DVT is a frequent and serious complication of IBD and can result in debilitating sequalae such as ulceration and pain. Data demonstrate that the traditional management of DVT with anticoagulation does not reduce the likelihood of PTS. This case series highlights that early intervention using thrombolysis with/without stenting is a safe and effective approach and should be considered as a treatment option in IBD patients with acute iliofemoral DVT after detailed evaluation of the risks. Patients need to receive detailed counselling regarding possible complications and potential benefits of the procedure. A multi-disciplinary approach with input from gastroenterology may improve outcomes.
